# Supplementary material for: Injection with Toxoplasma gondii protein affects neuron health and survival
Source: eLife. 2021 Jun 9;10:e67681. doi: 10.7554/eLife.67681 (PMC8270641; doi:10.7554/eLife.67681)
Supplement: Transparent reporting form [file elife-67681-transrepform.docx]

***eLife’s* transparent reporting form**

We encourage authors to provide detailed information *within their submission* to facilitate the interpretation and replication of experiments. Authors can upload supporting documentation to indicate the use of appropriate reporting guidelines for health-related research (see [EQUATOR Network](http://www.equator-network.org/%20)), life science research (see the [BioSharing Information Resource](https://biosharing.org/)), or the [ARRIVE guidelines](http://www.plosbiology.org/article/info:doi/10.1371/journal.pbio.1000412) for reporting work involving animal research. Where applicable, authors should refer to any relevant reporting standards documents in this form.

If you have any questions, please consult our Journal Policies and/or contact us: [editorial@elifesciences.org](mailto:editorial@elifesciences.org).

**Sample-size estimation**

- You should state whether an appropriate sample size was computed when the study was being designed
- You should state the statistical method of sample size computation and any required assumptions
- If no explicit power analysis was used, you should describe how you decided what sample (replicate) size (number) to use

Please outline where this information can be found within the submission (e.g., sections or figure legends), or explain why this information doesn’t apply to your submission:

A power analysis was not conducted to determine a sample size for Figure 1. Previous studies conducted in our lab have determined that we can find differences among groups with an N of 8-12 mice, as seen in Figure 1. For the electrophysiology section a chi-square test was conducted to measure differences between groups with at least 0.56 SD of difference, which corresponded to a medium effect size, at a significance level of 0.05 and with 0.8 power. For whole cell recordings, each cell was treated as independent samples.

**Replicates**

- You should report how often each experiment was performed
- You should include a definition of biological versus technical replication
- The data obtained should be provided and sufficient information should be provided to indicate the number of independent biological and/or technical replicates
- If you encountered any outliers, you should describe how these were handled
- Criteria for exclusion/inclusion of data should be clearly stated
- High-throughput sequence data should be uploaded before submission, with a private link for reviewers provided (these are available from both GEO and ArrayExpress)

Please outline where this information can be found within the submission (e.g., sections or figure legends), or explain why this information doesn’t apply to your submission:

For Figure 1, the experiment was replicated 4 times. Excluded animals from Figure 1 are represented in Supplemental Figure 1. Exclusion criteria are defined in materials and methods and in the relevant text. Figures 2,3 used brain sections from cohort 4 of Figure 1, except the sampling parameters were changed from Figure 1, which is described in materials and method (tissue preparation). For every figure, relevant details about sections/mouse and mouse/experiment are contained within the figure legends. For every figure except Figure 5, mean data for each individual mouse are identified.

**Statistical reporting**

- Statistical analysis methods should be described and justified
- Raw data should be presented in figures whenever informative to do so (typically when N per group is less than 10)
- For each experiment, you should identify the statistical tests used, exact values of N, definitions of center, methods of multiple test correction, and dispersion and precision measures (e.g., mean, median, SD, SEM, confidence intervals; and, for the major substantive results, a measure of effect size (e.g., Pearson's r, Cohen's d)
- Report exact p-values wherever possible alongside the summary statistics and 95% confidence intervals. These should be reported for all key questions and not only when the p-value is less than 0.05.

Please outline where this information can be found within the submission (e.g., sections or figure legends), or explain why this information doesn’t apply to your submission:

Statistical analysis details are contained within figure legends and in materials and methods (statistics). For Figure 1C, D, we reviewed the data with a biostatistician who advised using a one-sample t-test. Using a one-sample t-test allows us to identify an enrichment score of 1 as signifying random distribution, while deviation from 1 would indicate non-random distribution. For Figures 2 and 3, two-tailed t-tests were conducted to assess for difference between the two different *Toxoplasma* strains.

(For large datasets, or papers with a very large number of statistical tests, you may upload a single table file with tests, Ns, etc., with reference to sections in the manuscript.)

**Group allocation**

- Indicate how samples were allocated into experimental groups (in the case of clinical studies, please specify allocation to treatment method); if randomization was used, please also state if restricted randomization was applied
- Indicate if masking was used during group allocation, data collection and/or data analysis

Please outline where this information can be found within the submission (e.g., sections or figure legends), or explain why this information doesn’t apply to your submission:

Experimental groups are defined in the results and methods section. The experimenters were blinded for tissue processing and quantification of figures 1,2,3,7. For electrophysiology experiments experimenter is not blinded, since at time of recording GFP signal would be visible.

**Additional data files (“source data”)**

- We encourage you to upload relevant additional data files, such as numerical data that are represented as a graph in a figure, or as a summary table
- Where provided, these should be in the most useful format, and they can be uploaded as “Source data” files linked to a main figure or table
- Include model definition files including the full list of parameters used
- Include code used for data analysis (e.g., R, MatLab)
- Avoid stating that data files are “available upon request”

Please indicate the figures or tables for which source data files have been provided:

Source data will be provided for figures 1,2,3,5,6,7 is provided in the link: <https://osf.io/tqvzu/>

DOI 10.17605/OSF.IO/TQVZU
